# Psychotropic drugs interaction with the lipid nanoparticle of COVID-19 mRNA therapeutics

**DOI:** 10.3389/fphar.2022.995481

**Published:** 2022-09-09

**Authors:** Adonis Sfera, Sabine Hazan, Jonathan J. Anton, Dan O. Sfera, Christina V. Andronescu, Sarvin Sasannia, Leah Rahman, Zisis Kozlakidis

**Affiliations:** ^1^ Patton State Hospital, San Bernardino, CA, United States; ^2^ Department of Psychiatry, University of California, Riverside, Riverside, CA, United States; ^3^ Department of Biology, California Baptist University, Riverside, CA, United States; ^4^ Department of Anthropology, Stanford University, Stanford, CA, United States; ^5^ Shiraz University of Medical Sciences, Shiraz, Iran; ^6^ Department of Medicine, University of Oregon, Eugene, OR, United States; ^7^ International Agency For Research On Cancer (IARC), Lyon, France

**Keywords:** LNP, psychotropic drugs, cell-cell fusion, PEGylated lipids, DSPC, ionizable lipids, cholesterol analogs

## Abstract

The messenger RNA (mRNA) vaccines for COVID-19, Pfizer-BioNTech and Moderna, were authorized in the US on an emergency basis in December of 2020. The rapid distribution of these therapeutics around the country and the world led to millions of people being vaccinated in a short time span, an action that decreased hospitalization and death but also heightened the concerns about adverse effects and drug-vaccine interactions. The COVID-19 mRNA vaccines are of particular interest as they form the vanguard of a range of other mRNA therapeutics that are currently in the development pipeline, focusing both on infectious diseases as well as oncological applications. The Vaccine Adverse Event Reporting System (VAERS) has gained additional attention during the COVID-19 pandemic, specifically regarding the rollout of mRNA therapeutics. However, for VAERS, absence of a reporting platform for drug-vaccine interactions left these events poorly defined. For example, chemotherapy, anticonvulsants, and antimalarials were documented to interfere with the mRNA vaccines, but much less is known about the other drugs that could interact with these therapeutics, causing adverse events or decreased efficacy. In addition, SARS-CoV-2 exploitation of host cytochrome P450 enzymes, reported in COVID-19 critical illness, highlights viral interference with drug metabolism. For example, patients with severe psychiatric illness (SPI) in treatment with clozapine often displayed elevated drug levels, emphasizing drug-vaccine interaction.

## 1 Introduction

In 2021, the Centers for Disease Control and Prevention prioritized vaccination for mentally ill individuals as psychiatric illness was added to the list of COVID-19 risk factors (Mazereel et al., 2021). Currently, there are very few studies on mRNA vaccine efficacy in patients with SPI in treatment with psychotropic drugs. However, increased breakthrough infections and limited vaccine responses were reported by a recent epidemiological study on veterans with SPI, highlighting possible drug-vaccine interaction (Nishimi et al., 2022). This study is in line with earlier data, showing that, in general, patients with SPI exhibit suboptimal vaccine effectiveness, a phenomenon also documented in the geriatric population (Solomon et al., 1970; Hussar et al., 1971; Della Bella et al., 2007; Derhovanessian and Pawelec, 2012; Wang et al., 2016). Indeed, immunological similarities, but also differences, exist between the SPI patients and older individuals. For example, persons with SPI exhibit a shorter-than-average lifespan and high comorbidity with age-related diseases, implicating premature cellular senescence in this pathology (Lindqvist et al., 2015; Lee et al., 2021; Pousa et al., 2021). In addition, SPIs were associated with lower counts of regulatory T cells (Tregs) that are often reversed by the treatment with psychotropic drugs (Hwang et al., 2009; Laursen, 2011; Papanastasiou et al., 2011; Kelly et al., 2018; Solana et al., 2018; Corsi-Zuelli et al., 2021). On the other hand, unlike older individuals, SPI patients display an increased number of natural killer cell (NKC) that are unaffected by the psychotropic drugs, probably explaining the low prevalence of malignancy as well as COVID-19 critical illness in this population (Yovel et al., 2000; Bao et al., 2021; Tarantino et al., 2021). Indeed, immune malfunction may account for both limited vaccine responses and protection from COVID-19 critical illness in medicated SPI patients (Nishimi et al., 2022), (Sfera et al., 2021; Nemani et al., 2022). For example, upregulated NKCs may promptly eliminate not only virus-infected but also mRNA-transfected cells, disrupting translation at the ribosomal level as well as antibody production (Arai et al., 1983; Brieva et al., 1984; Mason et al., 1988). In addition, psychotropic drugs’ anti-inflammatory and immunosuppressant actions may protect against virus-induced “cytokine storm” but at the same time lower immune reactivity necessary for adequate vaccine responses (Gobin et al., 2014; Baumeister et al., 2016; Wei and Hui, 2022).

## 2 Messenger RNA vaccines

The novel mRNA COVID-19 vaccines were inspired by the similarity between extracellular vesicles (EVs) and liposomes, a characteristic exploited in the treatment of hereditary transthyretin-mediated amyloidosis, a therapy comprised of small interfering ribonucleic acids (siRNAs) embedded in LNPs (Antimisiaris et al., 2018; Urits et al., 2020). Replacing siRNA content with mRNA led to the concept of LNP therapeutics encoding for the SARS-CoV-2 spike (S) protein to elicit neutralizing antibodies against it (Manjunath et al., 2005; Suzuki and Ishihara, 2021). Compared to other methods of exogenous nucleic acid introduction into cells, such as viral vectors, LNPs are better tolerated, although their transfection efficacy is less robust (Settanni et al., 2022).

To effectively deliver the synthetic mRNA to host ribosomes, LNPs must avoid several obstacles, including hydrolysis by extracellular RNases, activation of intracellular immune sensors, and degradation by the enzymes of the endosomal lysosomal system (ELS) (Cullis and Hope, 2017) (Sahay et al., 2013; Maugeri et al., 2019). Modifying and hiding mRNAs in LNPs can overcome the first two barriers, while ionizable lipids SM-102 (Moderna) and ALC-0315 (Pfizer BioNTech) may conquer the last one (Hou et al., 2021).

The mRNA-containing LNPs are comprised of four lipids: 1,2-distearoyl-sn-glycero-3-phosphocholine (DSPC), PEG, an alternative cholesterol, and ionizable lipids SM-102 or ALC-0315 (Benne et al., 2018; Aldosari et al., 2021). The SM-102, ALC-0315, and the alternative cholesterol are proprietary molecules and have not been revealed up to the present time. However, interrogating siRNA platforms, it is reasonable to conclude that ionizable lipids may resemble DLin-MC3-DMA and that a phytosterol may replace the cholesterol (Tam et al., 2013; Xia et al., 2020).

LNPs enter cells by endocytosis or phagocytosis (immune cell uptake) (Birge et al., 2016) (Battistelli and Falcieri, 2020). Entry by the endocytic pathway (EP) can take place *via* clathrin-dependent or independent routes. Regardless of the ingress modality, LNPs travel from the early to late endosomes and can withstand an environmental pH of 5.5 or higher (Paliwal et al., 2015; Baranov et al., 2019). As exposure to the lysosomal pH of 4.5–5.0, could degrade the LNPs, endosomal-lysosomal system (ELS) exit must take place in the late endosomes (Paliwal et al., 2015). However, as late endosomes can also release their cargo *via* EVs, LNPs may be expulsed into the extracellular space instead of the cytosol (Gurung et al., 2021). Indeed, studies with split green fluorescence proteins (GFPs) have found that endosomal escape in general is an inefficient process as only about 2% of ELS content reaches the cytosol (Teo et al., 2021). This ratio can be increased with the help of negatively charged phospholipids, such as phosphatidylserine (PS) or analogs. For example, externalized PS (ePS) on ELS membranes generates an electrostatic imbalance between the cationic lipids and anionic phospholipids, enabling LNP to escape (Brock et al., 2019; ur Rehman et al., 2013; Wojnilowicz et al., 2019).

Taken together, the successful delivery of LNP to the host translation machinery depends on overcoming several key obstacles. A major bottleneck that must be successfully negotiated to ensure cargo delivery involves LNP lysosomal evasion as well as the premature expulsion into the extracellular compartment.

### 2.1 The lipid nanoparticles in the cytosol and potential drug-interactions

**FIGURE 1:**
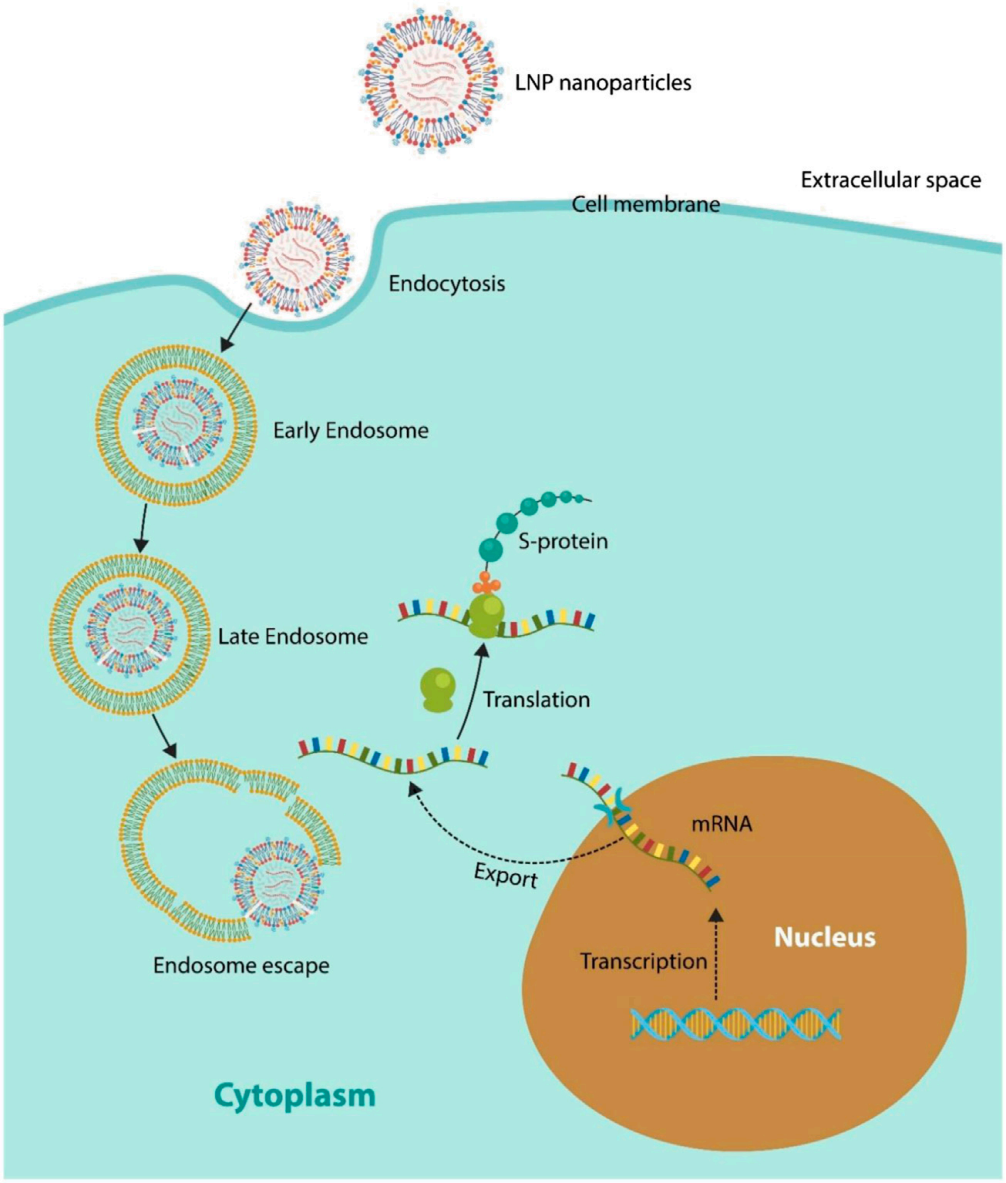
LNPs enter host cells *via* endocytosis or phagocytosis (immune cell endocytosis). LNP is trafficked through the ELS, traveling from early to late endosomes. Progressing from late endosome to lysosomes would risk LNP degradation by the hydrolyzing enzymes; therefore, ELS escape must take place in late endosome. However, late endosomes may expulse their cargo into the extracellular compartment *via* EVs (not shown). This is a major hurdle that LNPs must negotiate. Under ideal circumstances, ribosomes translate the exogenous mRNA into the S protein. For this to occur, it must be assumed that the human translation machinery does not differentiate between endogenous (nucleus-derived) and exogenous mRNA.

There is a paucity of studies discussing the fate of LNP-mRNA in the cytosol. It is generally assumed that once released from the liposome, mRNA can find its way to the ribosomes where the S antigen is translated (Wu and Li, 2021) (Figure 1). However, the modified vaccine mRNA may be perceived by the cell as defective or damaged, holding back translation by ribosomal stalling (Baker and Coller, 2006; Karamyshev and Karamysheva, 2018; Chandrasekaran et al., 2019; Kow et al., 2021; Brest et al., 2022; Deb et al., 2021). Interestingly, several psychotropic drugs, including aripiprazole, clozapine, and lithium, were demonstrated to alter ribosomal function and protein synthesis, highlighting possible interference with the vaccine mRNA (Bayraktar et al., 2021; Liu et al., 2022).

Single molecule tracking studies have found that cytosolic mRNA can access cellular cytoskeleton and to travel throughout the cytosol from where it can diffuse into cytoplasmic organelles, including the nucleus (Yamagishi et al., 2013) (Vargas et al., 2005) (Fusco et al., 2003) (Siwaszek et al., 2014). For example, a recent study found that upon entering the host nuclear compartment, Pfizer BioNTech mRNA could be retrotranscribed into DNA by the long interspersed nuclear element-1 (LINE-1), emphasizing vaccine-genome interactions (Zhang et al., 2021; Aldén et al., 2022). Interestingly, upregulated LINE-1, a marker of SPI, can be lowered by psychotropic drugs (*via* DNA methylation), likely reducing, or averting the transcription of RNA to DNA (Houtepen et al., 2016; Doyle et al., 2017) (Table 1).

**TABLE 1:** Psychotropic drugs compound some LNP effects, altering cell entry, endosomal release, and exit of mRNA vaccines and their responses.

LNP component	Cellular effects	Psychotropic drugs	Interactions	References
PEG	Entry *via* EP BBB permeability increase (LNP CNS entry)	Entry by EP, alter pH (Phenothiazines, pimozide)	-Delayed LNP cellular uptake	Wang et al., (1993); Daniel (2003); Inoue et al. (2007); Popova et al. (2013); Chang et al. (2014); Canfrán-Duque et al. (2016); Kovtun et al. (2020); Meyer et al. (2021)
			-Lower LNP endosomal escape	
DSPC	Lower immunity and inflammation	Lower immunity and inflammation	Lower neutralizing antibody formation	Gobin et al. (2014); Baumeister et al. (2016); Batista-Duharte et al. (2021); Galván-Peña et al. (2021); Leykin et al. (1997); May et al. (2019); Ponsford et al. (2019); Schleuning et al. (1989); Goldsmith (2002); Lozano et al. (2015); Himmerich et al. (2010); Ficarra et al. (2016)
Ionizable lipids	Alter membrane asymmetry Alter polyamine homeostasis	Alter membrane asymmetry (Chlorpromazine, Risperidone)	-Lower formation of antibodies	Himmerich et al. (2010); Ficarra et al. (2016); Soulet et al. (2004); Goldman et al. (2009); Li et al. (2017); Jiang et al. (2020); Ding et al. (2021); Soulet et al. (2004); Goldman et al. (2009); Li et al. (2017); Jiang et al. (2020); Ding et al. (2021); Soulet et al. (2004); Goldman et al. (2009); Li et al. (2017); Jiang et al. (2020); Ding et al. (2021); Eygeris et al. (2020); Sebastiani et al. (2021)
			-Polyamines exhibit antidepressant and anxiolytic effects	
Cholesterol analogs	Transport by ApoE Phytosterols connected to neurodegeneration	Upregulate ApoE Promote cholesterol egress	May compromise vaccine efficacy by increased cholesterol egress	Cenik et al. (2017); Balasubramanian et al. (2022); Dean et al. (2003); Korade et al. (2017); Luquain-Costaz et al. (2020) Dean et al. (2003); Korade et al. (2017); Luquain-Costaz et al. (2020)

EP, endosomal pathway; LNP, lipid nanoparticle; ApoE, apolipoprotein E; PEG, polyethylene glycol; DSPC, 1,2-distearoyl-sn-glycero-3-phosphocholine.

In the following sections, we take a closer look at the four LNP lipids and their interaction with psychotropic drugs.

#### 2.1.1 PEGylated lipids

PEGylated lipids extend the duration of mRNA action and facilitate LNP endocytosis, while lowering aggregation and opsonization during the circulation (Yang and Shen, 2006; Li et al., 2014). However, despite these advantages, PEGylation raises the so-called “PEG dilemma”: prolongation of both LNP uptake and ELS escape, risking vaccine-mRNA degradation by the lysosomal enzymes (Fang et al., 2017). Given that the LNP composition is proprietary, it is unknown how the mRNA-based vaccines overcome the “PEG dilemma” however, linking PEG oxygen to the head group of SM-102 or ALC-0315 is a previously documented solution (Park et al., 2021). In addition, CHARMM-GUI membrane builder (http://www.charmm-gui.org/input/membrane), an in-silico lipid simulation platform, highlights PEG oxygen bond as the likely overcomer of the “PEG dilemma” (Lee et al., 2019).

### 2.2 Psychotropic drugs and PEGylated lipids

Several classes of psychotropic drugs, including phenothiazines were demonstrated to inhibit the EP by binding to adaptor protein 2 (AP2), a cell membrane protein that plays a crucial role in clathrin-mediated endocytosis (CME) (Hussar et al., 1971; Inoue et al., 2007; Kovtun et al., 2020). As CME is a major LNP intake mechanism, psychotropic drugs-inhibited AP2 likely disrupt vaccine transfection (Wang et al., 1993; Chang et al., 2014). Moreover, psychotropic medications were demonstrated to accumulate in lysosomes (lysosomotropism) and increase the ELS pH, likely delaying LNP escape thus, lowering the vaccine efficacy (Daniel, 2003; Canfrán-Duque et al., 2016). Along this line, the antipsychotic drug pimozide was shown to disrupt the LNP exit from ELS, emphasizing a drug-vaccine interaction that can inactivate the mRNA vaccines (Popova et al., 2013; Meyer et al., 2021). Interestingly, PEGylated liposomes were previously shown to induce cytochrome P450, especially CYP3A1, CYP2C6, and CYP1A2, causing accelerated blood clearance (ABC), a phenomenon that reduces the efficacy of PEGylated nanocarriers, emphasizing a less discussed LNP weakness (Su et al., 2018; Liu et al., 2020).

#### 2.2.2 1,2-distearoyl-sn-glycero-3-phosphocholine

DSPC is a non-pyrogenic, neutral phospholipid that plays a key role in cell apoptosis and immune regulation (Chaurio et al., 2009). DSPC was added to the LNP to prevent immune detection by the cytosolic sensors, including toll-like receptors (TLRs) and retinoic acid-inducible gene I (RIG-I) (Zhang et al., 2022). DSPC plays a major role in stealthy LNP entry into the cells without alerting the host immune defenses. This is accomplished by altering the lipid asymmetry of plasma membranes, mimicking ePS, a global immunosuppressive signal (Birge et al., 2016). In addition, DSPC increases regulatory T cells (Tregs), further lowering host immune surveillance (Lin et al., 2006; Benne et al., 2020). Tregs upregulation is a double-edged sword as these lymphocytes can lower both virus-mediated inflammation (cytokine storm) and vaccine-evoked neutralizing antibodies, emphasizing that antiviral and pro-viral actions are highly intertwined (Batista-Duharte et al., 2021; Galván-Peña et al., 2021).

### 2.3 Potential interaction with psychotropic drugs

Psychotropic drugs may lower the robustness of vaccine responses by direct mechanisms, interaction with LNPs, or indirectly by the anti-inflammatory and anti-immunogenic properties of these agents (Baumeister et al., 2016), (Stapel et al., 2018; Lin et al., 2022). For example, the immunosuppressant properties of clozapine, haloperidol, risperidone, and antidepressant drugs are well-established, emphasizing likely interference with the vaccine-associated immunogenicity (Gobin et al., 2014) (Leykin et al., 1997; May et al., 2019; Ponsford et al., 2019). Indeed, biophysical studies show that antipsychotic drugs can insert themselves between the lipid molecules of plasma membrane, triggering anti-inflammatory responses that can impair vaccine efficacy (Jutila et al., 2001; Al-Amin et al., 2013; May et al., 2019; Pandurangi and Buckley, 2020). Moreover, leukopenia and decreased immunoglobulins, well-established properties of psychotropic drugs, may directly lower vaccine-elicited neutralizing antibodies (Ponsford et al., 2019) (Sherman et al., 1986; Goldsmith, 2002; Lozano et al., 2015). Interestingly, chlorpromazine, was found to also inhibit mRNA expression in human thymocytes, likely disrupting vaccine efficacy at the translation level (Schleuning et al., 1989) (Ficarra et al., 2016). Vaccine effectiveness can be further decreased by antipsychotic drugs-upregulated Tregs, an established defense mechanism against autoimmunity (Kelly et al., 2018) (Himmerich et al., 2010).

#### 2.3.3 Ionizable lipids

Ionizable lipids added to the LNP, SM-102 and ALC-0315, are pH-sensitive molecules, positively charged in an acidic environment and neutral at physiological pH (Paloncýová et al., 2021). This characteristic supports protonation, an event that facilitates LNP escape from the late endosomes (Maugeri et al., 2019), (Gao and Huang, 1996; Han et al., 2021).

Ionizable lipids likely contain synthetic polyamines as amine groups accumulate in the ELS, increasing membrane permeability that in turn promotes LNP transport into the cytosol (Figure 1) (Soulet et al., 2004; Goldman et al., 2009; Jiang et al., 2020). In addition, as polyamines play a key role in mRNA translation and stability, they may be key components of SM-102/ALC-0315 lipids (Li et al., 2017). Among the polyamines, spermine has demonstrated superior cellular uptake and endosomal escape ability, suggesting that LNPs may contain this molecule (Ding et al., 2021). In addition, spermine was shown to increase vaccine efficacy by upregulating autophagy in human T cells and enhancing antigen responses (Merkley et al., 2018; Alsaleh et al., 2020).

Novel studies attributed antipsychotic properties to spermine, while its dysfunction was associated with the pathogenesis of SPI, particularly suicidal behavior (Squassina et al., 2013; Yadav et al., 2018). Moreover, as spermine plays a major role in male and female reproductive physiology, disruption of this polyamine may contribute to infertility and decreased birth rates (Lefèvre et al., 2011). Indeed, epidemiological studies from several countries have reported lower 2021 natality rates compared to the previous year, as demonstrated by Italy (−9.1%), Spain (−8.4%), Portugal (−6.6%), and New York (−19.8%) that might reflect dysfunctional polyamine signaling (Aassve et al., 2021; McLaren et al., 2021). Although it is difficult to trace the source of any potential infertility to mRNA vaccines as dysfunctional polyamines were also documented in SARS-CoV-2 infection and several psychiatric disorders, it is important to investigate these biomolecules further (Fiori and Turecki, 2008; Zhao et al., 2008; Gross and Turecki, 2013; Bourgin et al., 2021; Firpo et al., 2021).

### 2.4 Potential interference with psychotropic drugs

Several psychotropic drugs were shown to alter the integrity of membrane phospholipids, suggesting possible interference with the LNP ingress and ELS escape (Daniel, 2003). In addition, psychotropic drugs were demonstrated to alkalinize the ELS that in turn could disrupt the pH-dependent polyamines (Canfrán-Duque et al., 2016). Moreover, accumulating evidence suggests that polyamines, including putrescine, spermidine, and spermine, are not only involved in the pathogenesis of SPI but are also modulated by the antipsychotic drugs, suggesting possible interference with the mRNA vaccines (Squassina et al., 2013) (Fiori and Turecki, 2008). For example, spermidine, a spermine derivative, was found protective of the GABAergic and dopaminergic systems, suggesting that LNPs may interfere with this signaling (Yadav et al., 2018). This is significant as dopamine is not only involved in psychiatric disorders but is also an established fertility promoter, and dopamine agonists are frequently prescribed as part of assisted reproduction technology (ART) (Heiczman and Tóth, 1995; Tang et al., 2016)*.*

#### 2.4.1 Cholesterol analog

The cholesterol analog utilized in LNP is likely a phytosterol, as these molecules display high transfection capability by binding to apolipoprotein E (ApoE), followed by rapid endocytosis (Eygeris et al., 2020; Sebastiani et al., 2021). However, phytosterols have a major disadvantage as they suppress phagocytosis, probably limiting the LNP uptake in immune cells and therefore, mRNA translation (Yuan et al., 2019; Guo et al., 2022). In addition, unlike cholesterol, phytosterols cross the blood–brain barrier (BBB) and accumulate in the brain where their oxidation may precipitate the development of neurodegenerative disorders (Gamba et al., 2015; Sharma and Tan, 2021).

Several sterols, including desmosterol, were associated with both major depressive disorder and antidepressant medication, possibly accounting for the rare post-vaccination psychiatric symptoms recorded in VAERs (Cenik et al., 2017) (Balasubramanian et al., 2022; Chen et al., 2022). Moreover, cholesterol and other sterols can interact directly with dopamine transporters (DAT), possibly accounting for the post-vaccination dyskinesia noted in some patients with Parkinson’s disease (Sharma and Tan, 2021) (Jones et al., 2012; Erro et al., 2021).

### 2.5 Potential interference with psychotropic drugs

Several psychotropic drugs, including clozapine, olanzapine, haloperidol, and imipramine, were shown to up-regulate ApoE, a cholesterol transporter disrupted in SPI, suggesting possible interference with LNP transfection (Barroso et al., 2015; Dean et al., 2003; Digney et al., 2005; Miatmoko et al., 2021; Vik-Mo et al., 2009). As psychotropic medications upregulate the ATP-binding cassette transporter A1 (ABCA1), increasing cholesterol egress from cells, a process that may compromise vaccine efficacy by flushing LNPs into the extracellular compartment prior to mRNA release (Luquain-Costaz et al., 2020). In addition, several psychotropic drugs, including aripiprazole, haloperidol, and trazodone, were reported to increase the levels of cholesterol precursor, desmosterol, that in turn upregulates the expression of cholesterol efflux genes, likely removing LNPs from cells prematurely (Korade et al., 2017).

## 3 Limitations

This study has potential limitations. Firstly, it refers to a new technology within the clinical standard of care, which even though has a considerable body of literature in the scientific and preparatory phases, it is still developing the breadth of scientific observations from a clinical perspective. Secondly, it is likely that some of the potential drug-immunization interactions in the latest pandemics might be masked by the vaccine escape properties attributed to newly emerging SARS-CoV-2 variants, and as such an even more careful approach of the subject would be required to distinguish these compounding factors of lower than anticipated immune efficacy. Thirdly, it is presumed that some of the above observed interactions would have similarities in the future provision of mRNA therapeutics for non-communicable diseases, such as different cancer types. However, this remains a working hypothesis that requires further testing.

## 4 Discussion and conclusion

We opine that more studies are needed to assess the interaction between the major classes of psychotropic drugs, including antipsychotics, antidepressants, and mood stabilizers with the mRNA therapeutics. As the polyethylene glycol (PEG) component of lipid nanoparticles (LNPs) increases the permeability of BBB for a short interval, we anticipate that LNPs will be rapidly adopted by neuropsychiatry as vehicles for drug transport and delivery to the select CNS networks. For this reason, it is important to develop a VAERS-like system for recording the interaction of psychotropic drugs with current and future mRNA therapeutics.

Up to date, the exact LNP composition has not been released therefore, we analyze earlier data and virtual screening research, attempting to “fill-in” the blanks. For this reason, our assumptions and evidence may seem circumstantial, however, we believe, provide a foundation worth of further investigation.

LNPs are crucial for transporting exogenous mRNA to the host translational machinery where the S antigen is synthesized, eliciting neutralizing antibodies. The four LNP lipids guide the mRNA-loaded particle through the maze of extra and intracellular compartments, releasing its cargo into the cytosol. However, several bottlenecks on this journey, including ingress failure, delayed ELS escape, or premature expulsion from cells, may lower vaccine efficacy.

Treatment with psychotropic drugs may decrease the mRNA vaccine effectiveness by lowering inflammation/immunogenicity, inhibiting virus/LNP endocytosis, delaying ELS escape, or directly downregulating neutralizing antibodies.
